# Supplementary material for: Total Fatty Acid Analysis of Human Blood Samples in One Minute by High-Resolution Mass Spectrometry
Source: Biomolecules. 2018 Dec 27;9(1):7. doi: 10.3390/biom9010007 (PMC6359376; doi:10.3390/biom9010007)
Supplement: Supplementary file 1 [file biomolecules-09-00007-s001.zip › Supplementary figures.docx]

**Figure S1**. Hydrolysis with ^18^O-labeled H_2_O improves the signal-to-background (S/N) for monitoring endogenous FA 16:0.

(**A**) Detection of deprotonated FA 16:0 (*m/z* 255.2335) in a blank sample after hydrolysis using sulfuric acid in acetonitrile/regular H_2_^16^O (9:1, V/V).

(**B**) Detection of deprotonated FA 16:0 (*m/z* 255.2335) in human plasma after hydrolysis using sulfuric acid in acetonitrile/regular H_2_^16^O (9:1, V/V). The S/N is calculated by normalizing the intensity of *m/z* 255.2335 in the plasma sample (panel B) to that of the blank sample (panel A)

(**C**) Detection of deprotonated FA 16:0 (*m/z* 257.2377 and *m/z* 259.2420) in a blank sample after hydrolysis using sulfuric acid in acetonitrile/H_2_^18^O (9:1, V/V).

(**D**) Detection of deprotonated FA 16:0 (*m/z* 257.2377 and *m/z* 259.2420) in human plasma after hydrolysis using sulfuric acid in acetonitrile/H_2_^18^O (9:1, V/V). The S/N is calculated by normalizing the sum of *m/z* 257.2377 and *m/z* 259.2420 intensities in the plasma sample (panel D) to that of the blank sample (panel C).

**Figure S2**. Hydrolysis with ^18^O-labeled H_2_O improves the signal-to-background (S/N) for monitoring endogenous FA 18:0.

(**A**) Detection of deprotonated FA 18:0 (*m/z* 283.2645) in a blank sample after hydrolysis using sulfuric acid in acetonitrile/regular H_2_^16^O (9:1, v/v).

(**B**) Detection of deprotonated FA 18:0 (*m/z* 283.2650) in human plasma after hydrolysis using sulfuric acid in acetonitrile/regular H_2_^16^O (9:1, v/v). The S/N is calculated by normalizing the intensity of FA 18:0 in the plasma sample (panel B) to that of the blank sample (panel A)

(**C**) Detection of deprotonated FA 18:0 (*m/z* 285.2692 and *m/z* 287.2736) in a blank sample after hydrolysis using sulfuric acid in acetonitrile/H_2_^18^O (9:1, v/v).

(**D**) Detection of deprotonated FA 18:0 (*m/z* 285.2685 and *m/z* 287.2731) in human plasma after hydrolysis using sulfuric acid in acetonitrile/H_2_^18^O (9:1, v/v). The S/N is calculated by normalizing the sum of *m/z* 285.2685 and *m/z* 287.2731 intensities in the plasma sample (panel D) to that of the blank sample (panel C).

A11

FAME 16:0 16:016:0

FAME 18:0 16:016:0

FAME 18:1(9)

B

**Figure S3**. Monitoring of endogenous FA 16:0 and FA 18:0 in human plasma (10 µl) by GC-MS analysis is hampered by poor signal-to-background.

**(A)** Monitoring of FAME 16:0 in human plasma (pink XIC of *m/z* 74) and a blank sample (black XIC of *m/z* 74). The panel on the right shows a zoom of the response from a reagent blank sample. The S/N is calculated by normalizing the intensity of FAME 16:0 (XIC) in the plasma sample (1.1e6) to that of the blank sample (3.3e4); S/N = 33 for analysis of 10 µl plasma. Analysis of 1 µl plasma would yield an S/N of approximately 3.3.

**(B)** Monitoring of FAME 18:0 in human plasma (pink XIC of *m/z* 74) and a blank sample (black XIC of *m/z* 74). The panel on the right shows a zoom of the response from the blank sample. The S/N is calculated by normalizing the intensity of FAME 18:0 (XIC) in the plasma sample (2.2e5) to that of the blank sample (1.2e4); S/N = 18 for analysis of 10 µl plasma. Analysis of 1 µl plasma would yield an S/N of approximately 1.8.


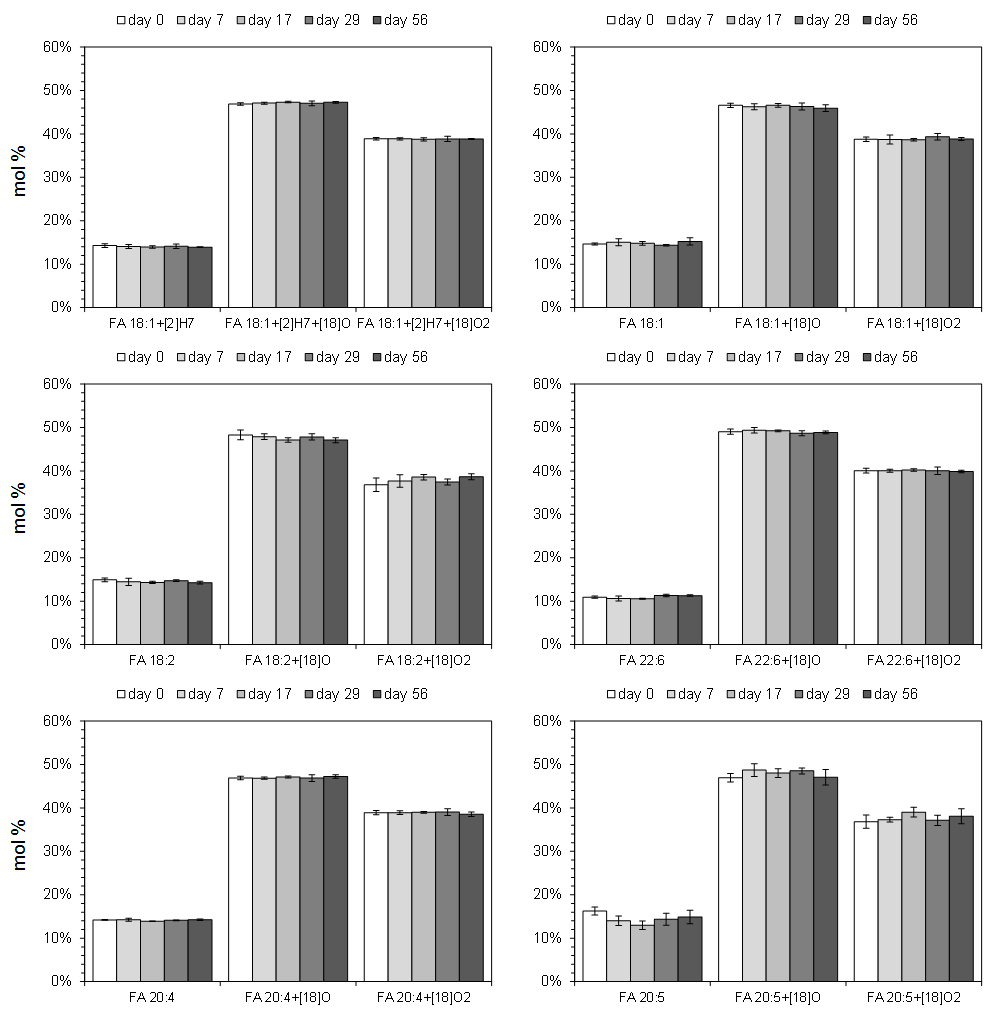


**Figure S4.** The incorporation of ^18^O is stable over time. Samples were hydrolyzed using sulfuric acid in acetonitrile/H_2_^18^O (9:1, v/v) and FAs were detected by FTMS analysis on the same day of the hydrolysis (day 0) and at days 7, 17, 29, and 56 after the hydrolysis. Relative intensities of different FA species containing two ^18^O, one ^18^O and one ^16^O, or only ^16^O are indicated.


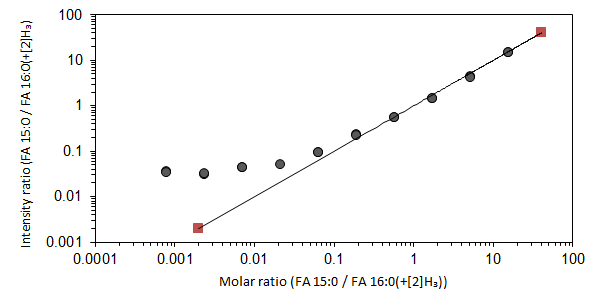


**Figure S5**. Dynamic range of FA 15:0 response. PE 15:0/18:1(+[2]H7) was titrated relative to a constant amount of LPC 16:0(+[2]H3), spiked into 1 µl human plasma and subjected to acid-catalyzed hydrolysis using H_2_^18^O. The total FA extracts were analyzed by direct infusion FTMS analysis. The x-axis shows the concentration of FA 15:0 relative to the concentration of FA 16:0(+[2]H3) (i.e., molar ratio). The y-axis shows the intensity of deprotonated FA 15:0 relative to the intensity of FA 16:0(+[2]H3). Depicted values derive from two replicate analyses. The line indicates the linear function with slope 1.

**Figure S6.** Total FA levels in plasma from normoinsulinemic and hyperinsulinemic subjects at 0 hr (baseline) and 6 hr after intake of FA 16:0(+[13]C16). Plasma lipids were subjected to acid-catalyzed hydrolysis with H_2_^18^O and analyzed by high resolution FTMS analysis. Data represent average ± SD of n=5 individuals.
