# Supplementary material for: Total Fatty Acid Analysis of Human Blood Samples in One Minute by High-Resolution Mass Spectrometry
Source: Biomolecules. 2018 Dec 27;9(1):7. doi: 10.3390/biom9010007 (PMC6359376; doi:10.3390/biom9010007)
Supplement: Supplementary file 1 [file biomolecules-09-00007-s001.zip › Table S1.docx]

**Table S1**. Accuracy for total FA analysis of SRM 2378 (human serum samples).

|  |  | **Fish oil** |  |  | **Flaxseed oil** |  |  | **No oil supplement** |  |
| --- | --- | --- | --- | --- | --- | --- | --- | --- | --- |
| Analyte | FTMS [µM] | NIST [µM] | Accuracy | FTMS [µM] | NIST [µM] | Accuracy | FTMS [µM] | NIST [µM] | Accuracy |
| FA 16:1 | 179 | 214 | 84% | 241 | 278 | 87% | 154 | 184 | 84% |
| FA 16:0 | 1897 | 3320 | 57% | 1844 | 2850 | 65% | 1487 | 2560 | 58% |
| FA 18:3 | 114 | 164 | 70% | 138 | 193 | 71% | 77.2 | 116 | 67% |
| FA 18:2 | 3177 | 3740 | 85% | 4041 | 4460 | 91% | 2885 | 3330 | 87% |
| FA 18:1 | 2154 | 2339 | 92% | 2779 | 2799 | 99% | 1961 | 2176 | 90% |
| FA 18:0 | 706 | 795 | 89% | 772 | 830 | 93% | 628 | 696 | 90% |
| FA 20:5 | 266 | 284 | 94% | 62.4 | 70.1 | 89% | 52.0 | 63.8 | 81% |
| FA 20:4 | 901 | 659 | 137% | 1200 | 790 | 152% | 1049 | 765 | 137% |
| FA 22:5 | 84.4 | 77.8 | 108% | 69.4 | 68.7 | 101% | 50.1 | 51.1 | 98% |
| FA 22:6 | 325 | 323 | 101% | 172 | 173 | 99% | 171 | 171 | 100% |
